# Evaluation of Factors Affecting Tree and Shrub Bark’s Antioxidant Status

**DOI:** 10.3390/plants11192609

**Published:** 2022-10-04

**Authors:** Nadezhda Golubkina, Ulyana Plotnikova, Vladimir Lapchenko, Helene Lapchenko, Sergey Sheshnitsan, Zarema Amagova, Visita Matsadze, Tatiana Naumenko, Natalia Bagrikova, Lidia Logvinenko, Tatiana Sakhno, Oksana Shevchuk, Nikolay Pirogov, Gianluca Caruso

**Affiliations:** 1 Analytical Laboratory Department, Federal Scientific Vegetable Center, 143072 Moscow, Russia; 2 T.I. Vyazemsky Karadag Scientific Station, Nature Reserve of RAS, 298188 Feodosia, Russia; 3 Department of Landscape Architecture and Soil Science, Voronezh State University of Forestry and Technologies, 394036 Voronezh, Russia; 4 Chechen Scientific Institute of Agriculture, 366021 Gikalo, Grozny Region, Russia; 5 Nikitsky Botanic Gardens, National Scientific Center of RAS, 298648 Yalta, Russia; 6 Bogdinsko-Baskunchak Nature Reserve, 416532 Akhtubinsk, Russia; 7 Department of Agricultural Sciences, University of Naples Federico II, Portici, 80055 Naples, Italy

**Keywords:** bark, *Cornus* and *Calligonum* species, antioxidant activity, polyphenols, stress factors

## Abstract

The importance of using the barks of trees and shrubs as powerful natural antioxidants suggests the necessity to evaluate the effect of different environmental factors on bark extracts’ quality. The determination of total antioxidant activity (AOA) and polyphenol content (TP) in the bark of 58 tree and shrub species from 7 regions differing in mean annual temperature, insolation, humidity, salinity level, and altitude was performed. The above stress factors positively affected bark AOA but did not have a statistically significant effect on TP. The bark of trees grown in the seashore proximity was characterized by significantly higher AOA than samples gathered in other areas, similarly to the trees grown at high altitude. The bark antioxidant status of 18 species was described for the first time. New sources of powerful antioxidants were represented by the ornamental shrubs *Cornus sanguinea* and *Cornus alba*, which showed the highest AOA (169–171 mg GAE g^−1^ d.w.). Among the typical halophytes, *Calligonum* and *Tamarix* had high AOA (172 and 85 mg GAE g^−1^ d.w.), while in the bark of tamarisk, an Se accumulator, an Se concentration of about 900 µg kg^−1^ d.w. was recorded. A significant positive correlation was found between leaves and bark AOA in the Karadag Nature Reserve’s deciduous trees (r = 0.898, *p* < 0.01). The relationship between bark AOA and TP was highly significant (r = 0.809; *p* < 0.001) for all samples except the mountainous ones. The results of the present research revealed new opportunities in successive bark utilization.

## 1. Introduction

Looking for new natural sources of powerful antioxidants, researchers have begun to pay more and more attention to tree wastes [1], and especially bark known to be byproducts of the wood processing industry. Bark wastes rich in polyphenols including tannins, lignin, and polysaccharides [2] compose about 20% of tree dry weight. Indeed, beech (*Fagus sylvatica* L., Fagaceae) contains up to 57 mg GAE g^−1^ d.w. of polyphenols in its bark [3,4,5], black poplar bark (*Populus nigra* L., Salicaceae) may accumulate up to 96.69–334.87 mg GAE g^−1^ of polyphenols [6], and willow bark is a well-known source of salicin [7].

A high polyphenols content suggests the prospects of bark utilization in the food industry, cosmetics, and herbal medicine [8]. The medicinal importance of tree bark is well documented for many tree species, revealing its antiinflammatory, chemopreventive, neuroprotective, cardioprotective, anticarcinogenic, antiviral, antibacterial, and antidiabetic effects [9,10,11]. Nevertheless, from a practical point of view it seems highly important to reveal factors affecting bark’s antioxidant quality. Nowadays, extremely scant information is available regarding factors affecting the accumulation of antioxidants in general and particularly polyphenols in tree bark. The age effect is an exception. Indeed, investigations relevant to the quantitative and qualitative composition of *Abies alba* Mill bark extracts revealed an increase in the total polyphenol extract yield and a significant decrease in phenolics diversity from the base to the top of the tree [12]. The relationship between tree age and bark antioxidant status was also reported for *Acacia confuse* [13] and *Cinnamomum loureirii* [14]. Special investigations were devoted to the effect of bark storage duration on antioxidants’ stability [15].

On the other hand, it is worth highlighting that a comparison of literature data is rather difficult due to the different solvents and duration related to the extraction, temperature regime, and variations in calculation methods, i.e., per dry weight of bark or dried extract.

The present investigation summarizes the data relevant to total antioxidant activity and polyphenol content in tree and shrub bark collected in seven different climatic regions varying by insolation, mean temperature, humidity, salinity level, and altitude.

## 2. Results and Discussion

### 2.1. Peculiarities of Antioxidants’ Distribution in Bark

**Figure 1:**
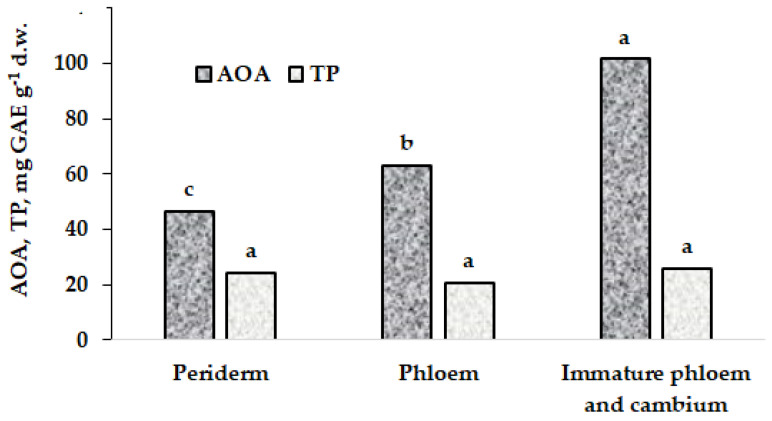
Antioxidants’ distribution between willow bark components. Values with the same letters do not differ statistically according to Duncan test at *p* < 0.05.

Metabolic processes determine plants’ antioxidant content to a large extent. Moreover, bark has a complicated anatomical structure and consists of different types of plant tissues [16]. An external part of bark is the periderm, a secondary protective tissue, which contains commonly protective cork (phellem) with nonliving cells heavily suberized at maturity. A middle part is the phloem with abundant sieve elements, which are a food conduit, and they must remain alive to provide their function. Moreover, bast fibers and sclereids with a lack of protoplast in cells at maturity as well as living parenchymal cells for temporal nutrient storage are included in the phloem. Finally, the internal part of bark is made up of immature phloem cells with a thin layer of meristematic tissue (cambium). All cells in the inner part of bark are actively metabolized to provide continuous cell division (cambium) or cell differentiation (immature phloem). Therefore, there should be significant differences in the antioxidant content between the three structural parts of bark. Figure 1 indicates willow antioxidants’ distribution, with the highest total antioxidant activity in the immature vascular tissue and cambium as well as significantly lower values in the phloem and periderm, while the total polyphenol content is similar in all three tissues.

This regularity of AOA decreasing from inner to external bark definitely corresponds with the metabolic activity of tissues and the proportion of live cells in them.

On the other hand, attention should be paid to the fact that even the lowest bark AOA values are higher or not different from the correspondent parameters recorded in popular vegetables such as parsley (AOA 44–68 mg GAE g^−1^ d.w.) [17], tomatoes (18–23 mg GAE g^−1^ d.w.) [18], and onion bulbs (34–58 mg GAE g^−1^ d.w.) [19], which proves the expedience of tree bark utilization as a significant source of natural antioxidants. The results presented below are mean values of antioxidant status for bark overall, including the cambium, phloem, and periderm due to difficulties separating these components from rather small tree branches.

### 2.2. Genetic Peculiarities of Antioxidants’ Accumulation in Bark

Experimental data of the total antioxidant activity (AOA) and phenolic content (TP) determination of tree and shrub bark from seven investigated regions indicated great differences in the parameters within a habitat (Table 1, Table 2, Table 3, Table 4, Table 5 and Table 6), which presents the first adequate comparison of 58 species’ antioxidant status due to similar conditions of extraction and analysis. Furthermore, despite numerous investigations of bark’s biological activity and antioxidant composition presented in citations (Table 1, Table 2, Table 3, Table 4, Table 5 and Table 6), it may indicate the lack of appropriate data for certain groups of species, which is expected for collection of trees in the Nikitsky Botanic Gardens (Table 4). Indeed, according to the monitoring results, special investigations are desirable regarding the bark biological activity and utilization of *Cornus sanguinea* and *Cornus alba, Picea pungens, Calligonum,* *Zanthoxylum americanum*, *Schinus lentiscifolius* and *molle*, *Laurus nobilis*, *Pinus gerardiena* and *bungeana*, *Vitex angus castus*, *Passilora caerulea*, *Platycladus orientalism*, *Styphnolobium japonicum*, *Ziziphus jujube*, *Morus alba*, *Ficus carica*, and *Prunus cerarifera*.

**Figure 2:**
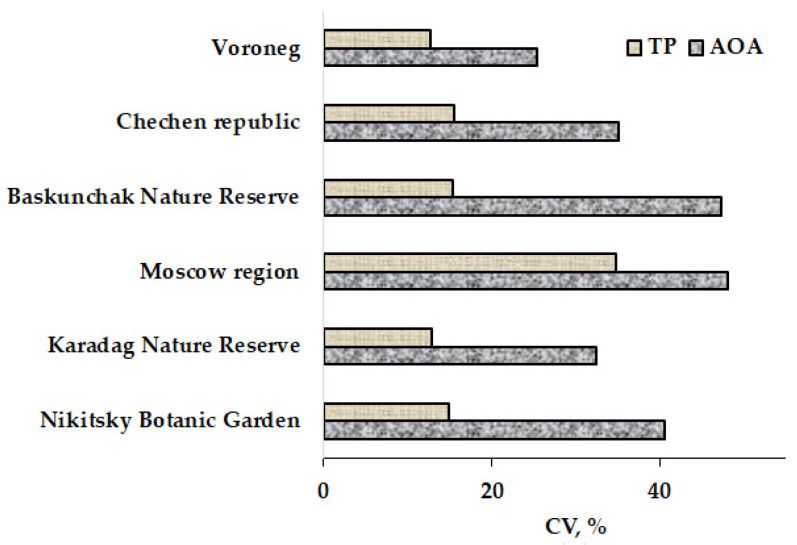
Coefficients of ecological variations (AOA and TP) calculated for tree bark.

According to the received data, the coefficient of ecological variation varied from 25.5 to 48.2% for AOA and from 12.9 to 34.9% for TP data, indicating high genetic variability in the tree barks’ antioxidant status (Figure 2).

The highest bark AOA levels were recorded for *Cornus sanguinea, Cornus alba*, and *Populus tremula* (162.5–170.0 mg GAE g^−1^ d.w.) in the Moscow region (Table 1); *Salix alba*, *Larix sibirica*, *Aesculus hippocastranum*, and *Corylus* (78–92 mg GAE g^−1^ d.w.) in the Voronezh region (Table 2); *Calligonum* (172 mg GAE g^−1^ d.w.) in the Bogdinsko-Baskunchak Nature Reserve (Table 3); *Zanthoxylum americanum, Schinus lentiscifolius, Schinus molle,* and *Myrtus communis* (98–115 mg GAE g^−1^ d.w.) in the Nikitsky Botanic Gardens (Table 4); *Armeniaca vulgaris*, *Cornus mas*, *Cotinus coggygria*, *Juglans nigra*, and *Aesculus hippocastanum* (100–119 mg GAE g^−1^ d.w.) in the Karadag Nature Reserve (Table 5), and *Acer campestre* and *Alnus* (102–118 mg GAE g^−1^ d.w.) in the Chechen republic (Table 6). Among the above-mentioned species, *Cornus sanguinea*, *Cornus alba*, *Calligonum*, *Zanthoxylum americanum*, *Schinus lentiscifolius*, and *Schinus molle* have never been characterized for bark antioxidant activity previously.

*Cornus sanguinea* and *Cornus alba* are of special interest and they are well-known decorative shrubs, the fruits of which are considered to be valuable tools as antidiabetic and hypoglycemic agents [67,68]. The quick growth and the practice of regular trimming make the bark utilization of these shrubs especially attractive.

As far as juzgun is concerned, its high content of *p*-coumaric acid in the stem [69] is in agreement with the present results, indicating the high prospects of its stem/bark utilization.

The wide collection of the Nikitsky Botanic Gardens’ plants gives the opportunity to reveal new natural sources of powerful antioxidants. In this respect, the high bark AOA of aromatic trees *Zanthoxylum americanum, Schinus lentiscifolius*, and *Schinus molle* may be considered as a good example of plants’ biodiversity. While *Zanthoxylum americanum* inhabits mostly Central and Eastern parts of the USA and Canada, *Schinus lentiscifolius* and *Schinus molle* are common to South America, Peru’s Andean deserts, Argentina, and Chile. Their quick growth, high tolerance to drought, and significant longevity have resulted in their fast distribution worldwide, and also as a serious invasive weed. To date, the bark of these trees has never attracted the attention of researchers and the present results indicate new approaches in terms of these trees’ utilization. The same situation is clear for myrtle bark, which has been used only for food and wine flavoring so far [49,70], and, therefore, its wider utilization should be developed.

Twice the lower variability of bark polyphenol content, compared to the AOA data, makes it more difficult to indicate the most valuable bark sources of these compounds. Nevertheless, there are high phenolics accumulations in the barks of *Armeniaca vulgaris, Cornus mas L., Cotinus coggygria, Zanthoxylum americanum, Schinus lentiscifolius, Salix, Populus tremula*, and *Alnus* (29–37 mg GAE g^−1^ d.w.). The latter characteristic is of great significance, taking into account the participation of phenolic compounds in regulating the immune system, and their several effects: antiinflammatory, chemoprevention, neuroprotection, cardio-protection, antidiabetes, Parkinson’s disease and cancer, and antibacterial [9,10] and as antivirals [11]. Variations in the phenolic and especially the flavonoid composition of bark is highly valuable for optimal bark extract utilization, such as in medicine, as preservatives in the food industry, in cosmetics, etc. [71,72,73], and it should be considered as the necessary step for further practice.

**Figure 3:**
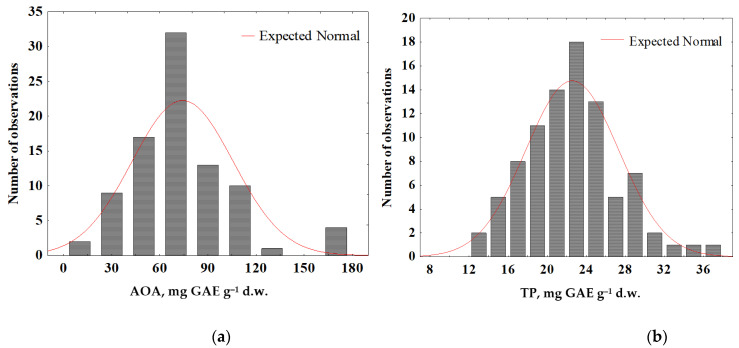
Histogram of total antioxidant activity (AOA) (**a**) and total polyphenols content (TP) (**b**) in tree bark of all species tested from all investigated regions (Nikitsky Botanic Gardens, Karadag Nature Reserve, Bogdinsko-Baskunchak Nature Reserve, Moscow region, Chechen republic, Voronezh region, Sovetskaya Gavan).

AOA and TP histograms of all species tested (Figure 3) show the normal distributions of these parameters with the median values reaching 72 and 23 mg GAE g^−1^ d.w., respectively.

**Figure 4:**
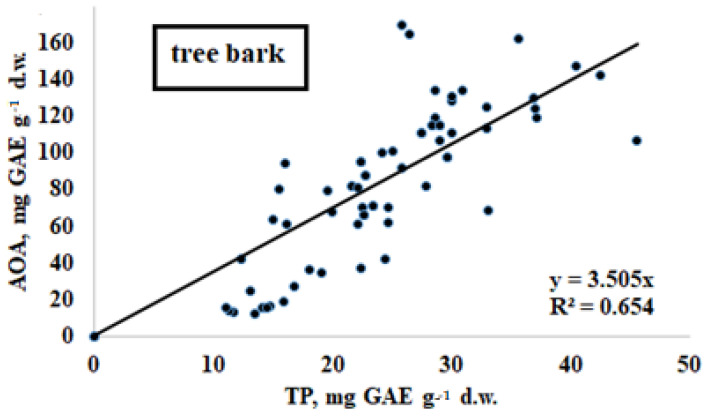
Relationship between AOA and TP in tree bark for all investigated regions except for Chechen Republic (r = 0.809; *p* < 0.001).

Many plant species demonstrate a significant positive correlation between their leaves’ total antioxidant activity and their polyphenol content [74], but, to date, this information has not been available for tree bark. The results of the present investigation indicate the existence of a linear significant relationship between these parameters for samples gathered in all regions, except the Chechen Republic (Figure 4). Obviously, the total antioxidant activity of bark is mainly determined by the polyphenol content not only in plant leaves but also in tree bark.

### 2.3. Effect of Altitude on Bark Antioxidant Status

**Figure 5:**
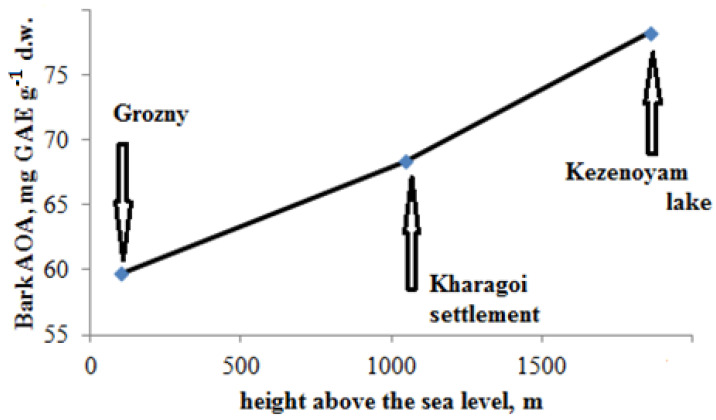
Effect of altitude on bark AOA status.

Bark sampling in the Chechen republic, situated in the northern part of Caucasus, gave an excellent opportunity to evaluate the altitude effect on bark AOA. In fact, differently from the other regions investigated, which were not so much affected by the altitude of bark sampling, the insignificance of the AOA/TP correlation in the Chechen Republic is supposedly connected to the altitude of bark sampling. Indeed, the mean bark AOA values for the plants of Grozny (103 m above sea level), Kharagol settlement (1008 m above sea level), and in the vicinity of the mountainous Kezenoyam lake (1800 m above sea level) differ significantly between each other, demonstrating that AOA increases with the altitude’s increase (Figure 5).

A similar relationship was described previously only for grasses [75] whose metabolism in general is quicker than bark’s biochemical changes. This phenomenon may be valuable in the production of bark extracts for the pharmaceutical industry in mountainous regions.

### 2.4. Salinity Effect on Bark AOA

Among the regions investigated, the Bogdinsko-Baskunchak Nature Reserve is characterized by high salinity due to the presence of a large salty lake (with the industrial production of salt), water deficiency, drought, and high solar insulation. These unfavorable conditions restrict flora diversity predominantly to grasses in most of the Reserve area. However, there is an area of experimental afforestation in a so-called ‘Green Garden’ with high soil permeability and significantly lower salt stress. Nevertheless, among the halophytes-residing areas with high salinity, two species, juzgun and tamarisk, are the most interesting (Table 3).

Both plant species are well known for their tolerance to salinity. All parts of tamarisk (leaves, florets, and bark) are widely used in the food industry and traditional medicine [43,76]. This species has previously been recorded as a powerful accumulator of selenium, a microelement with high antioxidant properties [74]. The determination of Se concentration in tamarisk bark revealed values up to 900 µg kg^−1^ d.w. The high biological activity of this plant may be partly related with the high levels of Se, a well-known natural antioxidant. Furthermore, investigations in the last years have indicated that the supplementation of food with tamarisk bark not only increases the antioxidant activity of products but during meat frying prevents the formation of toxic heterocyclic amines [77].

The literature data indicate the high prospects of juzgun utilization both in traditional medicine and the food industry due to its extremely high antioxidant activity [78]. The results of the present work are in agreement with the above data, indicating for the first time the antioxidant status not only of the whole aboveground shrub biomass but also of bark.

### 2.5. Effect of Seashore Vicinity

The comparison of the mean bark antioxidant status of trees for the seven regions investigated resulted in a lack of statistically significant differences between both the AOA and TP data (Table 7). On the other hand, reliable differences between the AOA parameters were recorded for median values between regions neighboring the seashore (Karadag Nature Reserve, Nikitsky Botanic Gardens, Sovetskaya Gavan) and intercontinental regions. Less pronounced differences in median values were recorded for TP values, indicating the Nikitsky Botanic Gardens’ plants as the most powerful sources of polyphenols.

**Figure 6:**
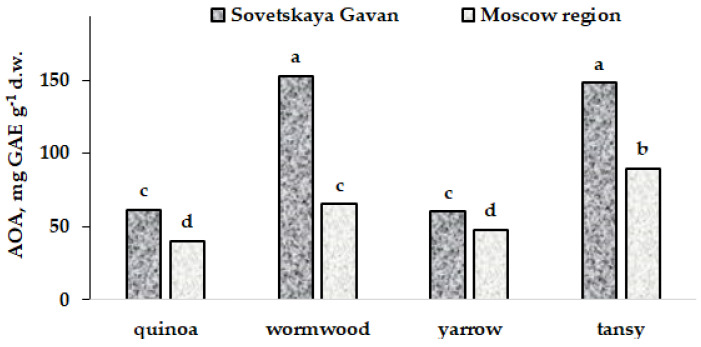
AOA of quinoa (*Atriplex*), yarrow (*Achillea millefolium*), tansy (*Tanacetum vulgare*), and wormwood (*Artemisia vulgaris*). Values with the same letters do not differ statistically according to Duncan test at *p* < 0.05.

Despite the small sample size in the Sovetskaya Gavan territory, associated with the presence of exclusively birch and separate apple trees on the coast of the Tatar Strait, the positive effect of seashore proximity on AOA was also confirmed by significantly higher levels of antioxidant activity in coastal grasses from Sovetskaya Gavan compared to similar data for the intercontinental region (Moscow region) (Figure 6).

The detected phenomenon of high bark AOA in the vicinity of the seashore has a complex character including the multiple effect of different stress factors: high levels of insolation, salinity, temperature, and humidity (in the case of the Sovetskaya Gavan coast). The intensive transfer of macro- and micro-elements from the sea surface supposes the possibility of additional stresses, which may stimulate the production of bark antioxidants. In any case, additional investigations are necessary to reveal the mechanism of the seashore proximity beneficial effect.

**Figure 7:**
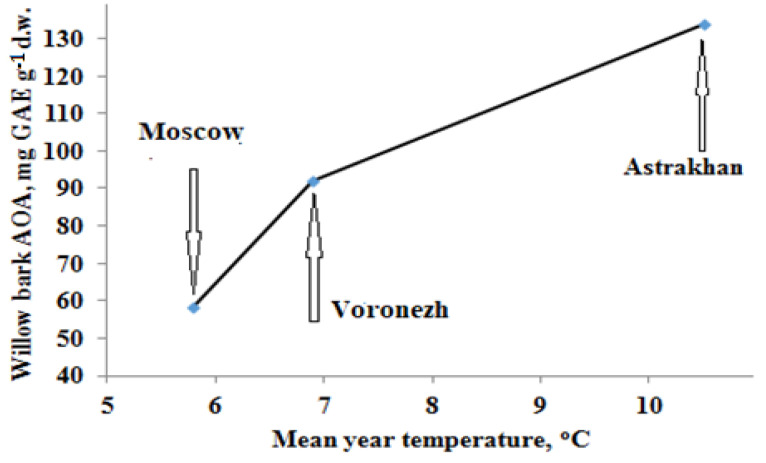
Geographical differences in AOA of willow bark.

Separate AOA data of bark samples gathered in regions with different temperature regimes support the beneficial effect of environmental stress (Figure 6). Indeed, willow bark showed the highest antioxidant activity in Astrakhan compared to the Moscow and Voronezh regions characterized by much lower mean annual temperatures (Figure 7).

### 2.6. Relationship between Leaves and Bark AOA

**Figure 8:**
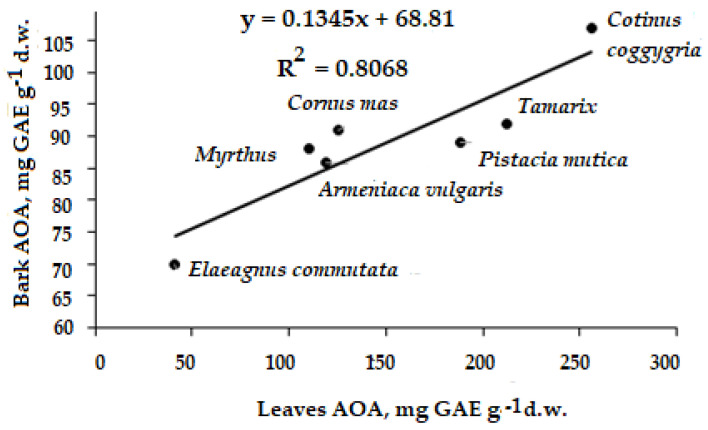
Leaves–bark AOA correlation for separate Karadag trees. (r = 0.898; *p* < 0.01).

Taking into account the incomparably higher life expectancy of the tree bark compared to the leaves of deciduous trees and the more intensive metabolism of leaves, a lack of correlation between the barks’ and leaves’ AOA can be expected. Nevertheless, we found a positive correlation between these parameters for seven species of the Karadag Nature Reserve (Figure 8). The results imply the prospects for leaves’ AOA determination for a quick search of trees with powerful bark AOA due to the significantly easier sampling of leaves.

In this respect, it may be supposed that there is a high bark AOA of *Vitis vinifera*, *Prunus armeniaca*, and *Quercus pubescens* grown in the Karadag Nature Reserve, with their leaves’ AOA values being in the range 81–86 mg GAE g^−1^ d.w. [74].

On the other hand, the lack of appropriate data for coniferous trees, greatly differing from the deciduous plants in terms of metabolism intensity, seed production methods, morphological peculiarities, and the existence of year-round photosynthesis, implies the necessity of further investigations, which may provide important information about the plants’ biology. Intermediate groups of species may be of special interest, such as larch and tamarack (*Larix* spp.), having needles and cones but also losing their leaves in the fall, or deciduous coniferous pond cypress (*Taxodium ascends*) and evergreen Rhododendron (Rhododendron spp.). Trees’ and shrubs’ diversity indicates multiple methods of plant adaptability, varying from the clear affiliation of trees to deciduous and coniferous groups to transitional forms combining the properties of both.

## 3. Materials and Methods

### 3.1. Object of Investigation

Bark sampling of 58 tree and shrub species was carried out in May–July 2022 (Table 8), using tree/shrub branches of 1–3 cm diameter. Bark samples were dried in an oven at 70 °C to constant weight and homogenized. Dry bark powder was used for the determination of total antioxidant activity (AOA) and total polyphenol content (TP).

### 3.2. Total Polyphenols (TP)

Total polyphenols in bark powder were determined in 70% ethanol (7:3, *v*/*v*) and water using the Folin–Ciocalteu colorimetric method as previously described [79]. One gram of dry homogenates was extracted with 20 mL of 70% ethanol/water (7:3, *v*/*v*) at 80 °C for 1 h. The mixture was cooled down and quantitatively transferred to a volumetric flask, and the volume was adjusted to 25 mL. The mixture was filtered through filter paper, and 1 mL of the resulting solution was transferred to a 25 mL volumetric flask, to which 2.5 mL of saturated Na_2_CO_3_ solution and 0.25 mL of diluted (1:1) Folin–Ciocalteu reagent (AppliChem Panceae, Darmstadt, Germany) were added. The volume was brought to 25 mL with distilled water. One hour later the solutions were analyzed on a spectrophotometer (Unico 2804 UV, Suite E Dayton, NJ, USA), and the concentration of polyphenols was calculated according to the absorption of the reaction mixture at 730 nm. As an external standard, 0.02% gallic acid (*w*/*w*) (Sigma-Aldrich, St. Louis, USA) was used. The results were expressed as mg of Gallic Acid Equivalent per g of dry weight (mg GAE g^−1^ d.w.).

### 3.3. Antioxidant Activity (AOA)

The antioxidant activity of samples was assessed using a redox titration method via titration of 0.01 N KMnO_4_ (analytical grade, Chimmed, Moscow, Rassia) solution with ethanolic/water extracts of dry samples [79], produced as described above. The reduction of KMnO_4_ to colorless Mn^2+^ in this process reflects the quantity of antioxidants dissolvable in 70% ethanol/water (7:3, *v*/*v*). The values were expressed in mg Gallic Acid Equivalents (mg GAE g^−1^ d.w.).

### 3.4. Selenium

Selenium in tamarisk was analyzed using the fluorometric method previously described for tissues and biological fluids [80]. Dried homogenized samples were digested via heating with a mixture of nitric and perchloric acids (analytical grade, Chimmed, Moscow, Russia), subsequent reduction of selenate (Se^+6^) to selenite (Se^+4^) with a solution of 6 N HCl, and formation of a complex between Se^+4^ and 2,3-diaminonaphtalene (Sigma-Aldrich, St. Louis, USA). Calculation of the Se concentration was achieved by recording the piazoselenol fluorescence value in hexane (analytical grade, Chimmed, Moscow, Russia) at λ = 519 nm emission and λ = 376 nm excitation. Each determination was performed in triplicate. The precision of the results was verified using a reference standard of Se-fortified chervil stem powder in each determination with an Se concentration of 1865 µg kg^−1^ (Federal Scientific Vegetable Center, Moscow, Russia).

### 3.5. Statistical Analysis

The data were processed by analysis of variance, and mean separations were performed through the Duncan’s multiple range test with reference to 0.05 probability level using SPSS software version 21. Data expressed as percentage were subjected to angular transformation before processing.

## 4. Conclusions

The present investigation reports for the first time the antioxidant statuses of 18 out of 58 studied trees and shrub species (*Cornus sanguinea* and *Cornus alba*, *Picea pungens, Calligonum,* *Zanthoxylum americanum, Schinus lentiscifolius* and *molle*, *Laurus nobilis*, *Pinus gerardiena* and *bungeana*, *Vitex angus castus*, *Passilora caerulea*, *Platycladus orientalism*, *Styphnolobium japonicum*, *Ziziphus jujube*, *Morus alba*, *Ficus carica*, and *Prunus cerarifera*), and indicate the significant genetic differences in the accumulation of polyphenols and total antioxidant activity of bark. Increased bark AOA has been recorded in areas close to the seashore and trees grown at high altitude. Other stress factors such as high salinity and temperature may also stimulate the accumulation of antioxidants in bark. Moreover, regardless of the habitat and species peculiarities, there exists a positive correlation between bark AOA and TP for trees grown at the same altitude. Furthermore, a positive correlation between leaf and bark AOA opens wide possibilities to valorize the quality of tree parts such as leaves and bark. Monitoring tree bark AOA allowed new powerful antioxidant-rich bark sources to be indicated: *Calligonum*, *Cornus sanguinea* and *Cornus alba*. Tamarisk bark showed not only high AOA but also high selenium accumulation. Further investigations are necessary to reveal other significant factors affecting the medicinal and nutritional qualities of tree bark.

## Figures and Tables

**Table 1:** Total antioxidant activity and phenolics content of tree and shrub bark in Moscow region.

Tree/Shrub Species	AOA, mg GAE g^−1^ d.w.	TP, mg GAE g^−1^ d.w.	References *
Deren, *Cornus sanguinea*	170.0 a	25.8 b	No data
Red Deren, *Cornus alba*	165.0 a	26.5 b	No data
Aspen, *Populus tremula*	162.5 a	35.7 a	[20,21]
Alder, *Alnus glutinosa*	119.0 b	37.2 a	[22]
Marple, *Acer platanoides*	94.2 b	16.0 d	[23]
Howthorn, *Crataegus* sp.	81.1 b	22.2 bc	[24]
Lilac, *Syringa vulgaris*	80.0 b	15.6 d	[25]
White willow, *Salix alba*	68.2 bc	33.1 a	[26]
Thuja, *Thuja occidentalis*	67.5 c	20.0 c	[27]
Joster, *Rhamnus* sp.	63.9 c	15.0 d	[28]
Rowan, *Sorbus* sp.	61.0 c	22.2 bc	[29]
Viburnum, *Viburnum* sp.	61.0 c	16.2 d	[30]
Ash-tree, *Fraxinus excelsior*	41.8 d	12.4 e	[31]
Linden, *Tilia cordata*	36.7 d	22.4 bc	[32]

* Citations devoted to bark’s biological activity; within each column, values with the same letters do not differ statistically according to Duncan test at *p* < 0.05.

**Table 2:** Total antioxidant activity and phenolics content of tree and shrub bark in Voronezh region.

Tree/Shrub Species	AOA, mg GAE g^−1^ d.w.	TP, mg GAE g^−1^ d.w.	References *
White willow, *Salix alba*	92.0 a	22.3 b	[26]
Larch, *Larix sibirica*	83.0 ab	30.0 a	[33]
Horse chestnut, *Aesculus hippocastranum*	79.0 ab	18.0 cd	[34]
Hazel, *Corylus avellana*	78.0 ab	17.2 cd	[35]
Prickly spruce, *Picea pungens*	74.0 b	18.2 cd	No data
Marple, *Acer campestre*	70.0 bc	24.7 ab	[23]
Ash, *Fraxinus excelsior*	69.1 c	16.8 d	[31]
Walnut, *Juglans regia*	68.0 cd	21.6 bc	[35]
Pine, *Pinus sylvestris*	65.0 cd	23.8 b	[36]
Poplar balsamic, *Populus balsamifera*	59.0 de	23.2 b	[37]
Oak, *Quercus robur*	59.2 de	20.1 bc	[38]
Black locust, *Robinia pseudoacacia*	53.0 e	20.9 bc	[39]
Marple, *Acer platanoides*	53.0 e	22.8 b	[23]
Birch, *Betula pendula*	42.0 f	24.4 ab	[40,41]
Small-leaved linden, *Tilia cordata*	40.0 f	19.2 cd	[32]
Elm, *Ulmus laevis*	36.1 f	20.9 bc	[32]

* Citations devoted to bark’s biological activity; within each column, values with the same letters do not differ statistically according to Duncan test at *p* < 0.05.

**Table 3:** Total antioxidant activity and phenolics content of tree and shrub bark in Bogdinsko-Baskunchak Nature Reserve.

Tree/Shrub Species	AOA, mg GAE g^−1^ d.w.	TP, mg GAE g^−1^ d.w.	References *
Juzgun, *Calligonum*	172.0 a	26.6 ab	No data
Willow, *Salix alba* **	134.0 b	26.5 ab	[25]
Siberian elm, *Ulmus pumila*	81.5 c	25.1 b	[42]
Tamarisk, *Tamarix*	76.0 cd	24.0 b	[43,44]
Lilac, *Syringa vulgaris*	73.6 cd	22.3 bc	[25]
Black pine, *Pinus nigra*	62.9 de	22.5 bc	[45]
Common oak, *Quercus robur*	60.5 e	21.9 bc	[46]
White poplar, *Populus alba*	47.5 f	19.0 c	[47]
Siberian peashrub, *Caragana arborescens*	43.8 f	31.9 a	[48]
Common ash, *Fraxinus excelsior*	20.0 g	20.2 c	[31]

* Citations devoted to bark’s biological activity; within each column, values with the same letters do not differ statistically according to Duncan test at *p* < 0.05. ** The sampling was performed in Astrakhan.

**Table 4:** Total antioxidant activity and phenolics content of tree and shrub bark in Nikitsky Botanic Gardens.

Tree/Shrub Species	AOA, mg GAE g^−1^ d.w.	TP, mg GAE g^−1^ d.w.	References *
Common pricklyash, *Zanthoxylum americanum*	115.0 a	28.4 a	No data
Schinus, *Schinus lentiscifolius*	115.0 a	29.0 a	No data
Schinus soft, *Schinus molle*	111.0 a	27.5 a	No data
Myrtle, *Myrtus communis*	98.0 a	29.7 a	[49]
Tamarisk, *Tamarix*	88.2 bc	22.3 b	[43,44]
Laurel noble, *Laurus nobilis*	71.1 cd	23.4 b	No data
Gerard’s pine, *Pinus gerardiana*	66.0 d	22.7 b	No data
Vitex sacred, *Vitex angus castus* L.	61.7 d	24.7 ab	No data
Bunge pine, *Pinus bungeana*	36.0 e	18.1 c	No data
Blue passionflower, *Passiflora caerulea*	33.9 e	28.7 a	No data

* Citations devoted to bark’s biological activity; within each column, values with the same letters do not differ statistically according to Duncan test at *p* < 0.05.

**Table 5:** Total antioxidant activity and phenolics content of tree and shrub bark in Karadag Nature Reserve.

Tree/Shrub Species	AOA, mg GAE g^−1^ d.w.	TP, mg GAE g^−1^ d.w.	References *
Common apricot, *Armeniaca vulgaris*	119.0 a	28.7 a	[50]
Common dogwood, *Cornus mas*	111.0 a	30.1 a	[51]
Tannery skumpia, *Cotinus coggygria*	107.0 ab	29.0 a	[52]
Black walnut, *Juglans nigra*	101.0 ab	25.1 ab	[35]
Horse chestnut, *Aesculus hippocastanum*	100.0 abc	24.2 b	[53]
Tamarisk, *Tamarix tetrandra*	92.1 bcd	25.9 ab	[43]
Strawberry tree, *Arbutus andrachne*	82.0 cde	27.9 ab	[54]
Pistachio, *Pistacia mutica*	79.0 de	19.6 c	[55]
Wolf-willow, *Elaeagnus commutata*	70.0 ef	22.6 c	[56]
Arbor vitae, *Platycladus orientalis*	70.2 ef	24.7 b	No data
Magnolia, *Mahonia aquifolium*	62.0 fg	22.4 c	[57]
High juniper, *Juniperus excelsa*	54.0 g	24.2 bc	[58]
Walnut, *Juglans regia*	43.0 h	23.3 c	[59]
Sophora japonica, *Styphnolobium japonicum*	34.1 j	20.0 c	No data

* Citations devoted to bark’s biological activity; within each column, values with the same letters do not differ statistically according to Duncan test at *p* < 0.05.

**Table 6:** Total antioxidant activity and phenolics content of tree and shrubs bark in the Chechen Republic.

Tree/Shrub Species	AOA, mg GAE g^−1^ d.w.	TP, mg GAE g^−1^ d.w.	References
Marple, *Acer campestre* ***	118.0 a	21.1 ab	[23]
Walnut, *Juglans regia* ***	83.3 bc	18.5 bc	[59]
Poplar, *Populus* sp. ***	64.6 d	18.6 bc	[60]
Birch, *Betula* sp. ***	63.2 d	17.5 bcd	[40,41]
Elder, *Sambucus* sp. ***	69.4 d	23.0 ab	[61]
Oak, *Quercus* sp. ***	67.1 d	21.2 ab	[38]
Willow, *Salix* sp. ***	59.3 de	21.2 ab	[26]
Unabi, *Ziziphus jujube* ***	57.0 def	20.2 bc	No data
Mulberry, *Morus alba* ***	49.1 f	17.3 bcd	No data
Linden, *Tilia* sp. ***	36.3 g	19.4 bc	[32]
Black locus, *Robinia pseudacacia* ***	29.6 h	21.1 a	[62]
Figs, *Ficus carica* ***	19.9 j	13.1 e	No data
Alder, *Alnus* sp. **	102.4 ab	25.8 a	[22]
Willow, *Salix* sp. **	89.5 b	21.4 a	[26]
Scots pine, *Pinus sylvestris* **	71.6 cd	25.0 a	[36]
Birch Radde, *Betula raddeana* **	67.9 d	18.9 bcd	[40,41]
Poplar, *Populus* sp. **	58.9 de	20.8 b	[60]
Wild medlar, *Mespilus germanica* *	86.6 b	24.5 a	[63]
Wild pear tree, *Pyrus* sp. *	79.3 d	17.0 cd	[64]
Wild apples tree, *Malus* *	73.3 bcd	17.4 bcd	[65]
Aspen, *Populus tremula* *	57.2 de	16.0 de	[20,21]
Wild walnut, *Juglans regia* *	56.3 ef	15.6 de	[59]
Wild prunes, *Prunus domestica* L. *	48.3 f	21.3 a	[66]
Wild cherry plum, *Prunus cerasifera* *	36.0 g	23.2 a	No data

* Kharagoi settlement: 1040 m above sea level; ** Kezenoiam lake: 1870 m above sea level; *** Grozny: 130 m above sea level; within each column, values with the same letters do not differ statistically according to Duncan test at *p* < 0.05.

**Table 7:** Geographical differences in AOA and TP of tree and shrub bark.

Region	AOA, mg GAE g^−1^ d.w.			TP, mg GAE g^−1^ d.w.		
	M ± SD	Conc. Range	Median	M ± SD	Conc. Range	Median
Nikitsky Botanic Gardens	76.7 ± 31.2 ab	24.2–115	82.0	25.5 ± 3.8 a	18.1–29.7	26.1
Karadag Nature Reserve	80.3 ± 26.1 ab	34.0–119.0	80.5	24.8 ± 3.2 a	20.0–30.1	24.5
Sovetskaya Gavan, Far East	89.2 ± 6.8 a	82.4–96.0	89.2	21.2 ± 0.7 a	20.4–21.9	21.2
Moscow region	84.8 ± 40.9 ab	36.7–170.0	68.2	22.9 ± 8.0 a	12.4–35.7	22.2
Bogdinsko-Baskunchak Nature Reserve	66.6 ± 31.6 ab	20.0–134.0	62.9	24.0 ± 3.7 a	19.0–31.9	23.3
Chechen Republic	64.3 ± 22.6 ab	19.9–118.0	63.9	20.0 ± 3.1 a	13.1–25.8	20.5
Voronezh region	60.1 ± 15.3 a	27.0–92.0	62.0	21.1 ± 2.7 a	16.8–30.0	20.9

Within each column, values with the same letters do not differ statistically according to Duncan test at *p* < 0.05.

**Table 8:** Tree species and places of bark sampling.

Region	Geographical Coordinates	n *	Tree/Shrub Species
Moscow region	55°39.51′ N, 37°12.23′ E	14	*Tilia cordata*, *Fraxinus excelsior*, *Viburnum* sp., *Sorbus* sp., *Rhamnus* sp., *Thuja occidentalis*, *Salix alba*, *Syringa vulgaris*, *Crataegus sanguinea*, *Acer platanoides*, *Alnus glutinosa*, *Populus tremula*, *Cornus alba*, *Cornus sanguinea*
Yalta, Nikitsky Botanic Gardens	44°30′73″ N, 34°14′09″ E	13	*Zanthoxylum americanum*, *Schinus lentiscifolius*, *Schinus molle*, *Myrtus communis*, *Tamarix tetrandra*, *Laurus nobilis*, *Pinus gerardiana*, *Pinus bungeana*, *Vitex agnus castus*, *Passiflora caerulea*
Karadag Nature Reserve	44°55′55″ N, 35°13′44″ E	14	*Armeniaca vulgaris*, *Cornus mas*, *Cotinus coggygria*, *Juglans nigra*, *Aesculus hippocastanum*, *Tamarix tetrandra*, *Arbutus andrachne*, *Pistacia mutica*, *Elaeagnus commutata*, *Platycladus orientalis*, *Mahonia aquifolium*, *Juniperus excelsa*, *Juglans regia*, *Styphnolobium japonicum*
Bogdinsko-Baskunchak Nature Reserve	48°11′00″ N, 46°53′00″ E	10	*Salix alba*, *Ulmus pumila*, *Tamarix ramosissima*, *Syringa vulgaris*, *Pinus nigra*, *Quercus robur*, *Populus alba*, *Caragana arborescens*, *Fraxinus excelsior*, *Calligonum aphyllum*
Chechen Rep. Kharachoy **	42°54′15″ N; 46°08′19″ E	6	*Mespilus, Pyrus* sp., *Malus* sp., *Prunus domestica*, *Prunus cerasifera*, *Juglans regia*
Chechen Rep. Kezenoiam lake ***	42°46′38″ N; 46°09′11″ E	6	*Alnus* sp., *Salix* sp., *Pinus sylvestris*, *Betula raddeana*, *Populus* sp., *Populus tremula*
Chechen Rep. Grozny ****	43°18′43″ N; 45°41′20″ E	12	*Acer campestre*, *Juglans regia*, *Populus* sp., *Betula* sp., *Sambucus* sp., *Quercus* sp., *Salix* sp., *Ziziphus jujube*, *Morus alba*, *Tilia* sp., *Robinia pseudacacia*, *Ficus carica*
Sovetskaya Gavan	48°57′59″ N 140°17′07″ E	2	*Malus* sp., *Betula* sp.
Voronezh region	51°40′18″ N 39°12′38″ E	18	*Salix alba*, *Larix sibirica*, *Aesculus hippocastanum*, *Corylus avellana*, *Picea pungens*, *Acer campestre*, *Fraxinus excelsior*, *Juglans regia*, *Pinus sylvestris*, *Populus balsamifera*, *Quercus robur*, *Robinia pseudoacacia*, *Acer platanoides*, *Tilia cordata*, *Ulmus laevis*, *Populus tremula*, *Betula pendula*

* n: number of species; ** height above sea level: 1008 m; *** height above sea level: 1800 m; ****—height above sea level: 103 m.

## Data Availability

Not applicable.
